# In Situ Observation of C−C Coupling and Step Poisoning During the Growth of Hydrocarbon Chains on Ni(111)

**DOI:** 10.1002/anie.202213295

**Published:** 2022-12-01

**Authors:** Zhiyu Zou, Alessandro Sala, Mirco Panighel, Ezequiel Tosi, Paolo Lacovig, Silvano Lizzit, Mattia Scardamaglia, Esko Kokkonen, Cinzia Cepek, Cristina Africh, Giovanni Comelli, Sebastian Günther, Laerte L. Patera

**Affiliations:** ^1^ CNR-IOM Materials Foundry Institute 34149 Trieste Italy; ^2^ Department of Physics University of Trieste 34127 Trieste Italy; ^3^ Elettra Sincrotrone Trieste 34149 Trieste Italy; ^4^ MAX IV Laboratory Lund University 22100 Lund Sweden; ^5^ Department of Chemistry and Catalysis Research Center Technical University of Munich 85748 Garching Germany; ^6^ Institute of Physical Chemistry University of Innsbruck 6020 Innsbruck Austria

**Keywords:** Fischer–Tropsch Synthesis, Heterogeneous Catalysis, Near-Ambient Pressure, Scanning Tunneling Microscopy

## Abstract

The synthesis of high‐value fuels and plastics starting from small hydrocarbon molecules plays a central role in the current transition towards renewable energy. However, the detailed mechanisms driving the growth of hydrocarbon chains remain to a large extent unknown. Here we investigated the formation of hydrocarbon chains resulting from acetylene polymerization on a Ni(111) model catalyst surface. Exploiting X‐ray photoelectron spectroscopy up to near‐ambient pressures, the intermediate species and reaction products have been identified. Complementary in situ scanning tunneling microscopy observations shed light onto the C−C coupling mechanism. While the step edges of the metal catalyst are commonly assumed to be the active sites for the C−C coupling, we showed that the polymerization occurs instead on the flat terraces of the metallic surface.

## Graphical abstract

The atomistic mechanisms driving hydrocarbon chain growth on a nickel model catalyst were elucidated with a combination of surface‐sensitive X‐ray photoelectron spectroscopy and in situ scanning tunneling microscopy. While the step edges of the catalyst are commonly assumed to be the active sites for C−C coupling, this research demonstrates that polymerization occurs on the flat terrace sites.

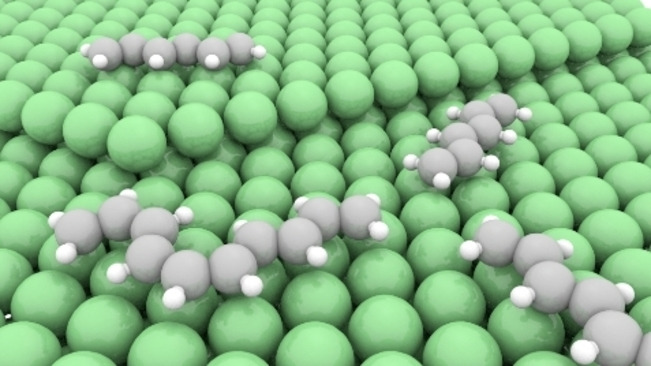

## Introduction

The polymerization of hydrocarbons into linear chains lies at the heart of many industrially relevant chemical reactions.[^1^, ^2^] One prominent example is the alkene polymerization with the Ziegler–Natta catalysts,[^3^, ^4^] which is responsible for two thirds of the global production of polyolefins.^[5]^ Long‐chain hydrocarbons can also be produced from syngas (a mixture of CO and H_2_) through the Fischer–Tropsch synthesis (FTS).[^6^, ^7^] This process is experiencing a renewed interest, especially in the context of modern power‐to‐gas and power‐to‐liquid plants.[^8^, ^9^, ^10^] The FTS is typically carried out on heterogeneous catalysts such as Co, Ru, and Fe.^[11]^ Ni has also proved to be active for the FTS, despite suffering from deactivation.^[12]^

From a microscopic point of view, the identification of the complex series of reaction steps involved in the process is still under debate.[^13^, ^14^] Surface‐sensitive techniques have proved to be an extremely powerful approach to explore the mechanisms of heterogeneous catalysis. However, due to the harsh reaction conditions of FTS (typically 500 K, 10–50 bar), in situ analysis using such techniques represents an experimental challenge.^[15]^ Recently, the Co(0001) surface exposed to syngas up to 4 bar at 500 K has been investigated by high‐pressure scanning tunneling microscopy (HP‐STM),^[16]^ where the step edges were identified as the active sites for the early stages of the FTS.^[17]^ At the final stage of the FTS, coupling of CH_2_ leads to the formation of linear oligomers with typical lengths up to tens of monomers.^[18]^ However, to date, little attention has been paid to the growth of hydrocarbon chains, and many open questions remain, in particular about the surface reaction pathways and the active sites for the hydrocarbon polymerization.

In an attempt to investigate the C−C bond formation step, ultra‐high vacuum (UHV) studies have been reported.[^13^, ^19^, ^20^, ^21^] Small hydrocarbon molecules were directly dosed on model catalyst surfaces,[^22^, ^23^] forming polymeric chains.[^24^, ^25^, ^26^] A recent in situ STM study clarified the growth process of polyethylene on a carburized Fe(110) surface, unraveling the ethylene insertion mechanisms proposed by Cossee for Ziegler–Natta catalysts.^[3]^ In this case, the active sites were identified as the boundaries between the iron carbide domains. Nevertheless, for other catalysts (e.g. Co)[^17^, ^27^, ^28^] the surface during the reaction is mostly metallic. Therefore, the actual active sites for the polymerization on such surfaces remain unclear. Though the step sites are also commonly assumed to catalyze the C−C coupling after being fully occupied by CH_*x*_ monomers,[^16^, ^29^] the typical strong adsorption of small molecules at the step edges[^30^, ^31^] could trap the CH_*x*_ species and thus hinder the polymerization. This behavior can be understood in the framework of the Sabatier's principle,^[32]^ stating that if the adsorption energy of the substrate is too low, then the catalytic activity is suppressed; if it is too large, then the product species will not desorb and block the surface, leading to catalyst poisoning. Therefore, elucidating the actual role of the step sites is crucial for an in‐depth atomistic understanding of the hydrocarbon chain growth process.

Here we investigated the growth of hydrocarbon chains on Ni(111) resulting from ethylene (C_2_H_4_) partial dehydrogenation into acetylene (C_2_H_2_) and subsequent polymerization. While X‐ray photoelectron spectroscopy (XPS) allowed the intermediate species and reaction products to be identified, in situ STM observations unveiled the C−C coupling step during the hydrocarbon polymerization. Notably, even though the step edges of the metallic surfaces are commonly assumed to be the active sites for the C−C coupling, we clarify that in the present case the polymerization occurs instead on flat terraces. Further insights obtained exploiting near‐ambient pressure X‐ray photoelectron spectroscopy (NAP‐XPS) at pressures up to 1 mbar revealed that the observed growth mechanism of hydrocarbon chains can be extended towards more industrially relevant conditions.

## Results and Discussion

**Figure 1:**
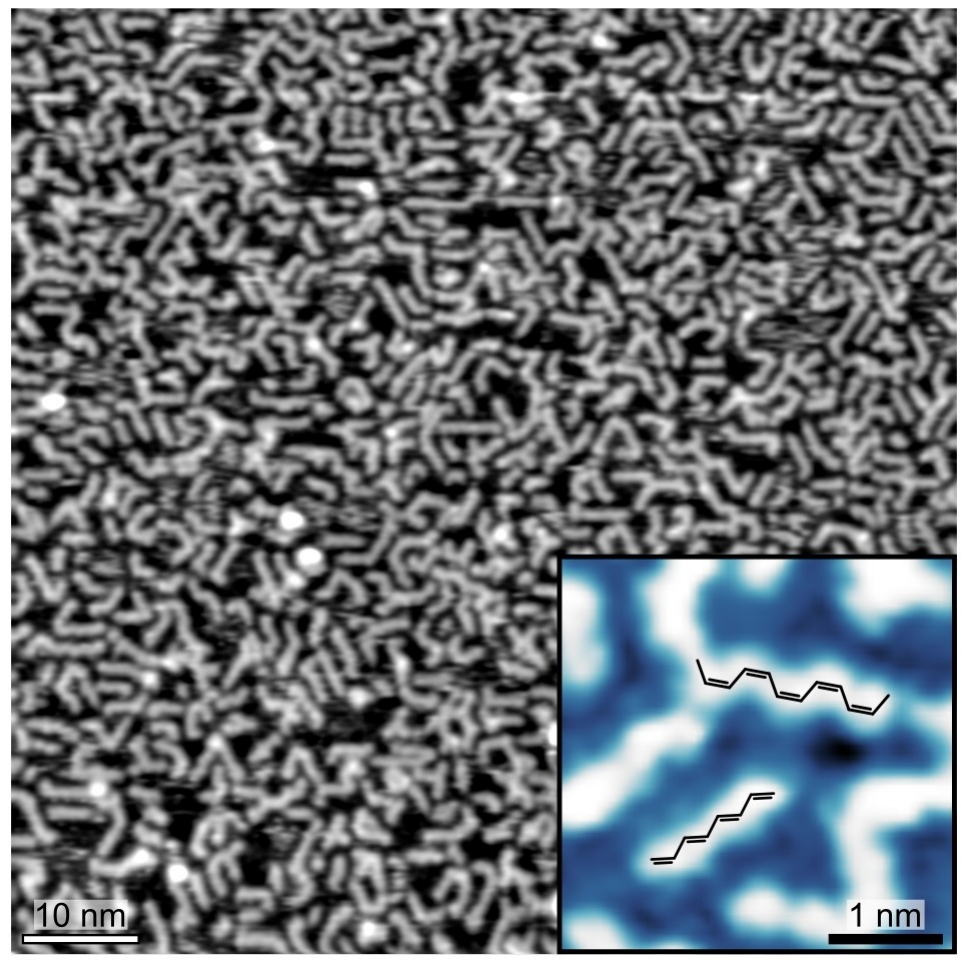
STM image showing on‐surface synthesized linear hydrocarbon chains on Ni(111), after ethylene exposure (1800 L, *t*=60′, *p*=5×10^−7^ mbar, *T*=343 K). Measurement parameters: tunneling current *I*=200 pA, sample bias voltage *V=*−0.3 V. Inset: high‐resolution STM image showing intramolecular details of the polymers (*V*=50 mV; *I*=1 nA). The overlaid models highlight *cis* and *trans* segments.

**Figure 2:**
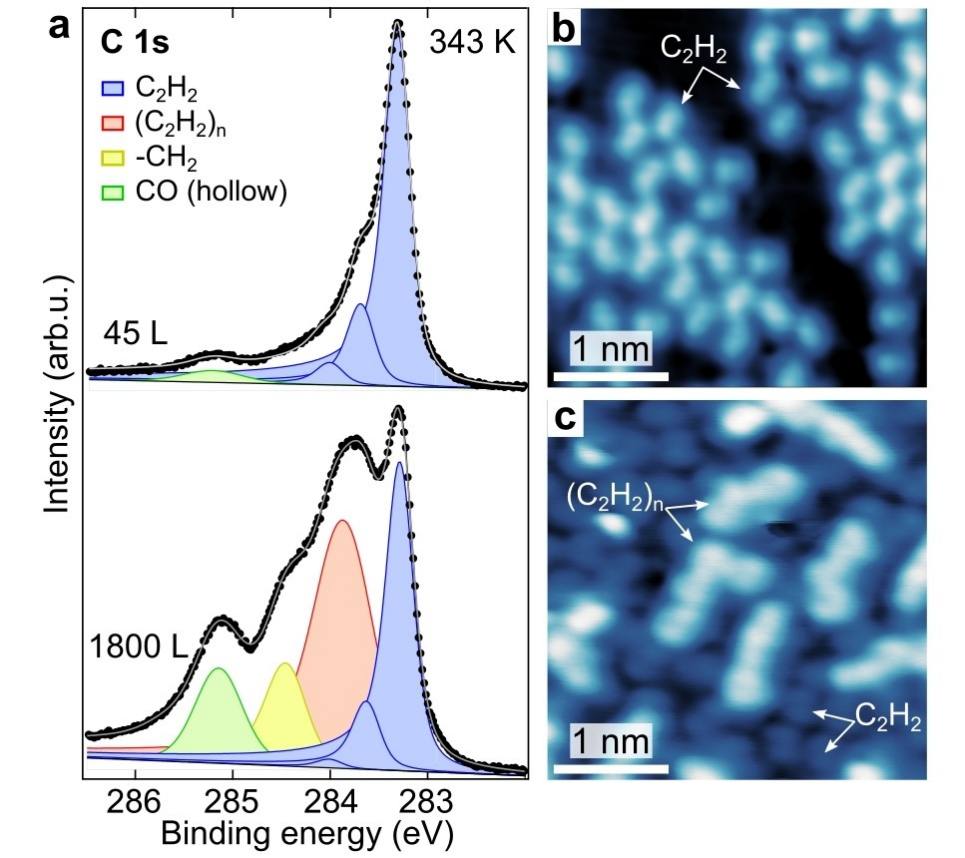
Growth of hydrocarbon chains upon ethylene partial dehydrogenation into acetylene and subsequent polymerization on Ni(111). a) High‐resolution XP spectra of the C 1s core level after dosing 45 L (top) and 1800 L (bottom) of ethylene at 343 K. Individual components obtained by the fitting procedure are superimposed as solid peaks. Photon energy=400 eV. b, c) LT‐STM images acquired after dosing 45 L and 1800 L of ethylene at 343 K, respectively. Functionalized STM tips were used to achieve sub‐molecular resolution. (b, *V*=0.2 V; *I*=0.1 nA; *T*=78 K), (c, *V*=50 mV; *I*=4.0 nA; *T*=78 K).

Figure 1 shows a rather dense layer of short‐chain hydrocarbons, obtained upon exposing Ni(111) to 1800 L of ethylene (C_2_H_4_, pressure *p*=5×10^−7^ mbar) at a temperature *T* of 343 K. A close‐up view of the polymeric structures manifests a periodicity of ≈0.25 nm and defined bending angles of 120°, suggesting the presence of alternating single and double bonds (see inset in Figure 1 and discussion below). To unveil the chemical nature of these structures, synchrotron‐based XPS was performed at the SuperESCA beamline of Elettra.^[33]^ Figure 2a (top) shows the C 1s spectrum acquired after exposing the Ni(111) surface to 45 L of ethylene at 343 K. A main peak at 283.3 eV is observed, together with two features at higher binding energies, shifted by ≈350 meV and ≈700 meV, respectively. An additional component at 285.1 eV is due to CO adsorption, coming likely from the chamber background pressure. The main peak corresponds to the C_2_H_2_ adiabatic transition, while the other components are attributed to transitions to excited vibrational states, with the energy splitting corresponding to the C−H stretching mode.^[34]^ This result is in agreement with the previously reported partial dehydrogenation of ethylene producing adsorbed acetylene between 200 K and 400 K on Ni(111)[^34^, ^35^] and Co(0001).^[20]^ Due to the strong electronic hybridization with the substrate,^[36]^ the adsorbed acetylene species are not expected to exhibit unpaired electrons. Dosing at a lower temperature (300 K), a small coverage of ethylidyne species (282.9 eV) is observed, and it disappears above 320 K (Figures S2 and S3), likely due to conversion into acetylene. Low‐temperature STM (LT‐STM) images acquired after the same sample preparation reveal the presence on the Ni(111) surface of adsorbed acetylene molecules with three distinct orientations (Figure 2b), reflecting the substrate symmetry.[^37^, ^38^, ^39^] These observations support the identification of the reactants as flat‐lying acetylene species.

At a much larger ethylene exposure (>600 L, *p*=5×10^−7^ mbar, see Figure S4), additional XPS components were clearly observed at 283.9 eV and 284.5 eV (Figure 2a, bottom), which saturate after ≈3000 L. LT‐STM image reveals the presence of acetylene monomers, co‐adsorbed with the elongated chains (Figure 2c) observed in Figure 1. The above results indicate that the two newly arising XPS components can be ascribed to the formation of polyacetylene oligomers (C_2_H_2_)_n_, as also supported by the intensity decrease of the acetylene's peak upon chain growth, pointing towards a polymerization process (Figure S4). The two C 1s components at 283.9 eV and 284.5 eV (Figure 2a, bottom) can be attributed to C atoms within the formed chains and to the passivated end of the oligomer chains (possibly CH_2_), respectively. This assignment is confirmed by comparison of XPS and STM data, since the intensity ratio between the two XPS components is 3.0±0.2, in rather good agreement with the length distribution obtained from STM images, being peaked at ≈4 units (see Figure S1).[^40^, ^41^] The broadening of the two XPS components of the hydrocarbon chains with respect to the ones of acetylene is ascribed to the multiple adsorption sites of the chains (Figure 1).

Recent studies carried out on Co(0001) revealed the role of CO as promoter for the transformation of acetylene into the ethylidyne and 2‐butyne.^[21]^ These results showed that the presence of co‐adsorbed CO alters the reaction products, leading to the CO‐induced hydrogenation of adsorbed acetylene‐producing ethylidyne. In our experiments, it was not possible to completely exclude the role of CO as a promoter for the hydrocarbon polymerization, as CO is found to adsorb from the chamber background pressure even under UHV conditions (see Figure 2a).

Although several studies concerning the investigation of the ethylene/Ni(111) system under UHV conditions have been reported[^30^, ^34^, ^42^, ^43^]—showing that upon adsorption partial dehydrogenation of ethylene occurs around 300 K and leads to the formation of acetylene species—no evidence of polyacetylene chains has yet been reported. We attribute this to the rather narrow parameter window required for the chain growth to take place. Around 300 K, a large exposure to ethylene (>600 L) is required to observe a significant coverage of polymers, while above 370 K the process competes with nickel carbide formation (Figure S2).^[44]^

**Figure 3:**
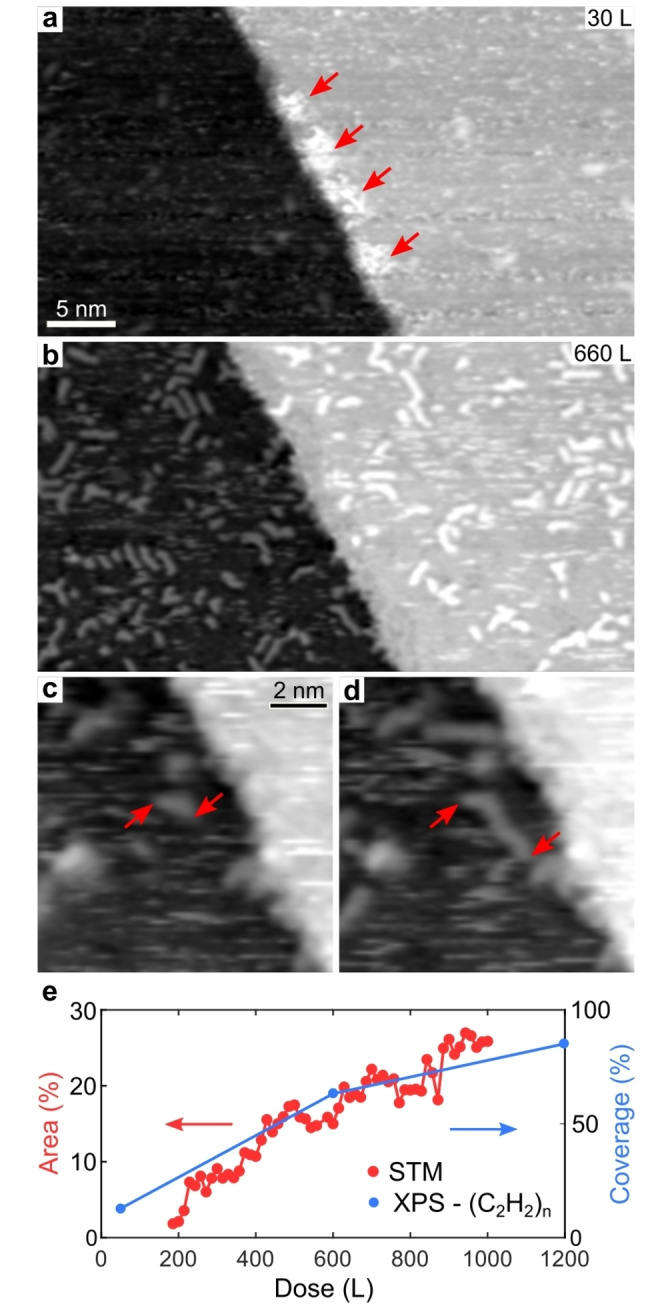
In situ STM studies of the polyacetylene oligomer growth acquired after a) 1 minute and b) 22 minutes of ethylene exposure (*V*=−0.1 V; *I*=0.2 nA; *T*=343 K; *p*=5×10^−7^ mbar). a) Arrows indicate the brim at step edge. c, d) Two consecutive STM images (38 s/frame). The sequence shows the growth of a polymeric chain (the chain's termini are highlighted by red arrows) occurring on the Ni(111) terrace. e) Evolution of the oligomer coverage extracted from the STM time series (pixel areas, red) and XP spectra (area of the XPS component at 284.5 eV in Figure S4, scaled to its saturation coverage as 100 %). The lateral size of the images used for the statistical analysis is ≈30×50 nm^2^. Note that STM and scaled XPS data curves coincide connecting the maximum covered surface fraction to the saturation coverage.

To elucidate the mechanism responsible for the polyacetylene chain growth, we performed in situ STM studies (see the movie in the Supporting Information). Figure 3a shows an STM image acquired after 60 s (30 L) of ethylene exposure (*p*=5×10^−7^ mbar) at 343 K. Short dashes are evident on the terraces, attributed to acetylene molecules rapidly diffusing on the surface due to the elevated temperature.[^45^, ^46^] At the step edge a brim is observed (see arrows), which has been previously assigned to dissociated hydrocarbon species.^[30]^ After a few minutes of continuous ethylene exposure, hydrocarbon chains are clearly observed in the STM sequence. Figure 3b shows a frame acquired after 660 L exposure, where a considerable fraction of the surface is covered by polyacetylene oligomers. Notably, once the chains are formed, they do not break, indicating covalent bonding. Even though surface diffusion typically prevents the clear imaging of the early stages of the chain formation, Figure 3c,d shows two consecutive STM images, where the growth of a chain can be observed. In general, the growth of polymeric chains can occur following two distinct routes: the step growth and the chain growth mechanisms.^[40]^ According to the step growth, polymerization occurs through a coupling reaction between two preformed chains. Instead, in the chain growth, the oligomer propagation proceeds by subsequent attachment of individual monomers. Despite the observation of chain propagation in Figure 3c,d, the detailed growth mechanism cannot be directly determined, mainly due to the limited time resolution of STM (38 s/frame). Nevertheless, indirect insight about the growth process can be obtained from a statistical analysis of the STM time series. Figure S1 shows the length distribution obtained from 414 chains. Notably, the experimental data resembles an Anderson–Schulz–Flory (ASF) distribution, where monomers and polymers are assumed to have equal reactivity at every stage. The good match of the experimental length distribution with the one derived from the ASF model suggests that the hydrocarbon polymerization proceeds by a step‐growth mechanism.

Figure 3e shows a plot of the oligomer coverage extracted from the STM sequence (see the Supporting Information for details on the analysis). The growth rate is in good agreement with the one extracted from the XPS data performed under comparable experimental conditions (see the (C_2_H_2_)_n_ component in Figure S4), supporting also the identification of the XPS components at 283.9 eV and 284.5 eV as hydrocarbon chains. The time evolution of surface coverage extracted from the STM time series exhibits an apparent initial induction time (Figure 3e). Apart from the screening of the STM tip,^[47]^ this effect can be ascribed to the fact that highly diffusing surface species cannot be clearly tracked by STM, even though they contribute to the XPS signal.

Taking a closer look at the STM time series (see the movie in the Supporting Information), a remarkable conclusion can be drawn about the active sites for the C−C coupling. At variance with what is commonly assumed,^[16]^ polymerization does not proceed by chain formation at the substrate step sites, but occurs almost exclusively on the flat terraces. At step sites, hydrocarbon chains form, but remain anchored and therefore do not detach. The observed inactivity of the step sites for the growth of polyacetylene oligomers is attributed to a strong binding of the hydrocarbon fragments, which prevents their release, causing self‐poisoning.[^30^, ^31^] This effect drastically reduces the turnover number of the step sites, resulting in a negligible catalytic activity.

The direct observation of the on‐terrace polymerization process (Figure 3) suggests the participation of native metal adatoms in the on‐surface catalytic process, as evidenced during graphene growth on Ni(111) and postulated during other on‐surface polymerization reactions,[^46^, ^48^, ^49^, ^50^, ^51^, ^52^], whichwill trigger theoretical investigations in the future.

**Figure 4:**
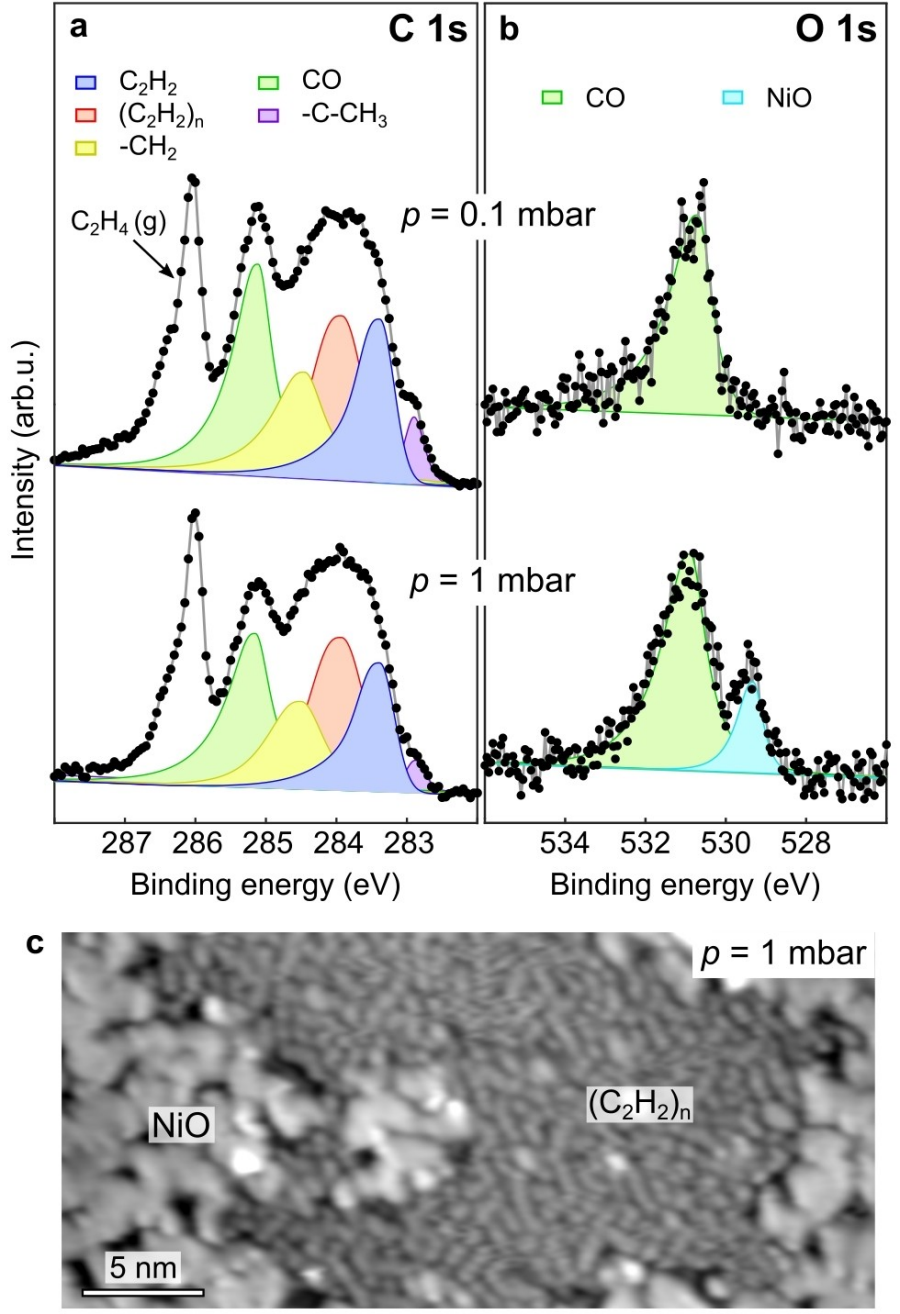
Hydrocarbon chains at near‐ambient pressures. a, b) NAP‐XP spectra of C 1s and O 1s core levels, respectively, acquired during ethylene dosing at *p*=0.1 mbar (top), and *p*=1 mbar (bottom) at 300 K. Photon energies: 400 eV (a) and 650 eV (b). The ethylene gas‐phase peak is indicated. c) STM image acquired after exposure of a clean Ni(111) to ethylene in a high‐pressure cell (*V*=−0.7 V; *I*=0.2 nA; *T*=300 K; *p*=1 mbar).

Finally, we addressed the extension of the presented growth process towards industrial conditions. For this purpose, near‐ambient pressure XPS (NAP‐XPS) has been performed at the SPECIES beamline of MAX IV.^[53]^ Figure 4a (top) shows the C 1s spectrum acquired at 300 K during exposure of the Ni(111) surface to ethylene at *p*=0.1 mbar. By comparison with the XP spectra acquired at lower pressures (Figure 2a), the fitting procedure reveals components for acetylene and hydrocarbon chains located at similar binding energies. The main differences lie in the presence of a small fraction of ethylidyne, and a significantly larger coverage of CO. While the former is likely due to the lower temperature (300 K, instead of 343 K in Figure 2), which stabilizes ethylidyne (Figure S2), the latter comes from the background pressure of the NAP chamber (*p*≈1×10^−8^ mbar) and the sticking coefficient of CO on Ni(111).^[54]^ Upon increasing the ethylene pressure to 1 mbar, the oligomer coverage increases. While at 0.1 mbar, only CO has been detected in the O 1s spectrum (Figure 4a), increasing the pressure of ethylene to 1 mbar leads to the formation of Ni oxide (Figure 4b).^[55]^ As imaged by UHV‐STM after exposure to 1 mbar, NiO patches appear as disordered islands (Figure 4c).^[56]^ According to previous studies, CO dissociation on Ni(111) can be excluded as a source of oxygen.[^57^, ^58^] Therefore, the oxide formation is attributed likely to oxygen impurities in the ethylene gas cylinder. Notably, the hydrocarbon chains can be clearly identified by STM imaging performed after the high‐pressure treatment (Figure 4c), demonstrating that the polyacetylene formation occurs also under NAP conditions, even in the presence of surface contaminants. Our findings show that the observed growth mechanism has the potential to be extended towards more industrially relevant conditions.

## Conclusion

In summary, we investigated the growth of hydrocarbon chains resulting from the polymerization of acetylene on a Ni model catalyst. While high‐resolution XPS provided chemical identification of the surface species, in situ STM elucidated the growth process, revealing the nature of the active sites. Although the step edges of the metal catalyst are commonly assumed to be active for the C−C coupling, we have shown that the polymerization occurs exclusively on flat terraces. Complementary studies at near‐ambient pressures show products of the on‐surface reaction that bear a remarkable resemblance, indicating that the self‐poisoning of the step edges of the catalyst observed under UHV conditions is valid up to the millibar pressure regime. As the Ni(111) and Co(0001) surfaces exhibit similar catalytic properties in promoting C−C coupling and hydrocarbon dehydrogenation,[^20^, ^21^] the reported acetylene growth mechanism could be relevant also for Co‐based catalysts.

## Conflict of interest

The authors declare no conflict of interest.

1

## Supporting information

As a service to our authors and readers, this journal provides supporting information supplied by the authors. Such materials are peer reviewed and may be re‐organized for online delivery, but are not copy‐edited or typeset. Technical support issues arising from supporting information (other than missing files) should be addressed to the authors.

Supporting Information

Supporting Information

## Data Availability

The data that support the findings of this study are available from the corresponding author upon reasonable request.
