# Supplementary material for: In Situ Observation of C−C Coupling and Step Poisoning During the Growth of Hydrocarbon Chains on Ni(111)
Source: Angew Chem Int Ed Engl. 2022 Dec 1;62(1):e202213295. doi: 10.1002/anie.202213295 (PMC10108169; doi:10.1002/anie.202213295)
Supplement: Supplementary file 1 — Supporting Information [file ANIE-62-0-s002.pdf]

Angewandte Chemie
Eine Zeitschrift der Gesellschaft Deutscher Chemiker

Supporting Information

In Situ Observation of C–C Coupling and Step Poisoning During the Growth of Hydrocarbon Chains on Ni(111)

*Z. Zou, A. Sala, M. Panighel, E. Tosi, P. Lacovig, S. Lizzit, M. Scardamaglia, E. Kokkonen, C. Cepek, C. Africh, G. Comelli, S. Günther, L. L. Patera**

Experimental Procedures

The STM experiments were carried out in two UHV systems ($p < 2\times10^{-10}$ mbar), equipped with standard sample preparation facilities. Ni(111) single-crystal samples were cleaned by several sputtering (Ar^+, 2 keV) and annealing (873 K) cycles. High-temperature experiments were performed with an Omicron VT-STM operated with a R9plus controller (RHK Technology). Low-temperature experiments were carried out with an Omicron Low-Temperature STM, working at ~ 78 K. Images were acquired in the constant current mode, with the bias voltage applied to the sample and the tip at ground. Electrochemically etched tungsten tips were used for imaging. The coverage plot shown in Figure 3e of the main text was derived by identifying the polymer chains as elongated particles in a particle analysis of the STM data by applying iterative threshold levelling (Igor Pro image package - particle analysis). After identification of each particle together with its individual area, the surface fraction covered by polymer chains displayed in Figure 3e can be easily calculated. Note that the STM data suffer from some noise due to tip changes which alters the threshold levelling outcome of the analysis and due to coverage fluctuations of diffusing species in- and outside the region of interest of the rather small imaged area.

The UHV-XPS experiments were performed at the SuperESCA beamline of the synchrotron radiation source Elettra (Trieste, Italy).[1] The Ni(111) crystal was mounted on a manipulator capable of providing fast-rate sample heating and cooling. The crystal was fixed to the cryostat by means of a Ta stick spot-welded on the back and was heated by W filaments placed behind the sample. High-resolution C 1s core level spectra were measured after cooling the sample to 80 K, at a photon energy of 400 eV, providing an overall energy resolution below 50 meV. For each spectrum, the binding energy was calibrated by measuring the Fermi level position of the Ni substrate. The measurements were performed with the photon beam impinging at grazing incidence (70°), while photoelectrons were collected at normal emission angle. The core level spectra were best fitted with Doniach–Šunjić functions convoluted with Gaussians and a linear background. The hydrocarbon chains were grown by dosing ethylene at pressures from 1×10^{-8} mbar to 5×10^{-7} mbar onto the Ni(111) surface kept at temperatures between 300 and 350 K. The C 1s binding energies of the CO peak, of the adiabatic and vibrational features of acetylene, as well as their intensity ratio have been restricted for the fit. The binding energy and the peak width of the polymer peaks were determined from a spectrum acquired at large coverage and then restricted for the uptake series.

The NAP-XPS experiments were performed at the SPECIES beamline of the MAX IV synchrotron (Lund, Sweden).[2] As sample, Ni(111) films grown on top of yttria-stabilized zirconia (YSZ)/Si(111) have been used. Several sputtering (1 keV, Ar^+) and annealing (873 K) cycles were performed in the preparation chamber. After transfer to the analysis chamber, the sample cleanliness was checked at a sample temperature of 300 K. C 1s and O 1s core level spectra were measured at a photon energy of 400 eV and 650 eV, respectively, with an overall energy resolution of approximately 200 meV. For each spectrum, the binding energy was calibrated by measuring the Fermi level position of the Ni substrate. For the measurements in near-ambient pressure conditions shown in Figure 4a-b, the sample-nozzle distance has been adjusted between the XP spectra at 0.1 mbar and 1 mbar, to optimize the surface signal against the gas phase one. The gas pressure was measured by a gas-independent manometer on the outlet line of the ambient pressure cell.

Results and Discussion

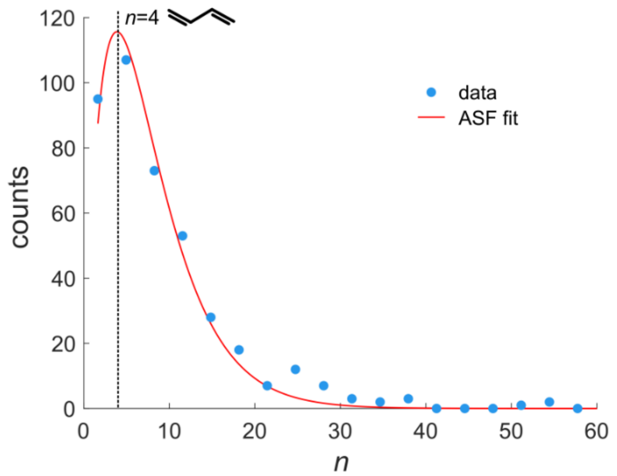

Figure S1. Length distribution of hydrocarbon chains. The number of carbon atoms constituting the hydrocarbon chains is defined as *n*. The statistics is done over 414 chains from an STM image shown in Figure 1 (80x80 nm^2) acquired after ethylene exposure (t = 60', p = 5×10^{-7} mbar, T = 343 K, dose = 1800 L). Red line represents a fit according to the Anderson-Schulz-Flory (ASF) distribution, giving a chain growth probability of about 77%.[3,4] The chemical structure of the most probable chain length (n = 4) is shown. The good match of the experimental length distribution with the one derived from the ASF model suggests that the hydrocarbon polymerization proceeds by a step-growth mechanism.

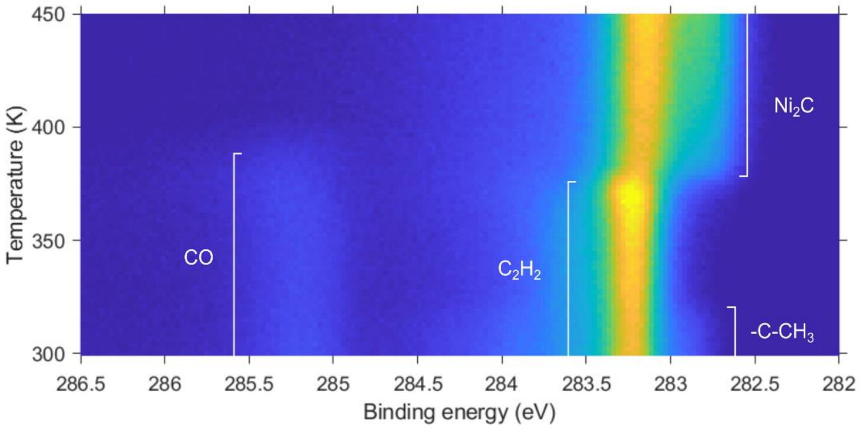

Figure S2. Temperature-programmed XP spectra of the C 1s core level acquired in UHV after dosing 20 L of ethylene at 300 K on Ni(111). Temperature ramp = 0.5 K/s, photon energy= 400 eV. The stability temperature ranges for the adsorbed species are indicated. A small coverage of ethylidyne can be observed around 300 K, which disappears at 320 K. The adsorbed acetylene species are found stable on the Ni(11) up to 380-390 K.

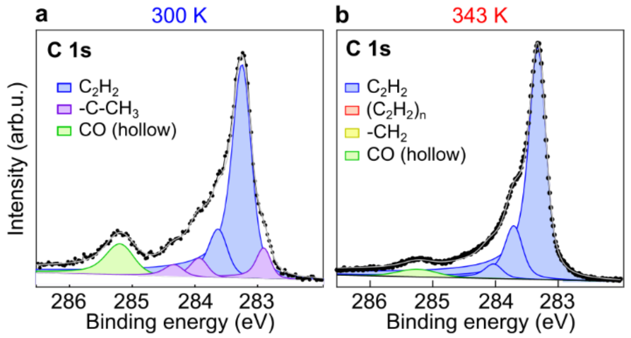

Figure S3. Ethylidyne (-C-CH_3) stability. a,b) XP spectra of the C 1s core level after dosing 20 L at 300 K (a) and 45 L at 343 K (b). Photon energy = 400 eV. a) Note that the spectral overlap of the peaks from acetylene and ethylidyne between 283.0 eV and 284.5 eV hinders a quantitative deconvolution. However, the component at lower binding energy (282.9 eV) is a clear fingerprint of ethylidyne. b) Spectrum reproduced from Fig. 2a (top).

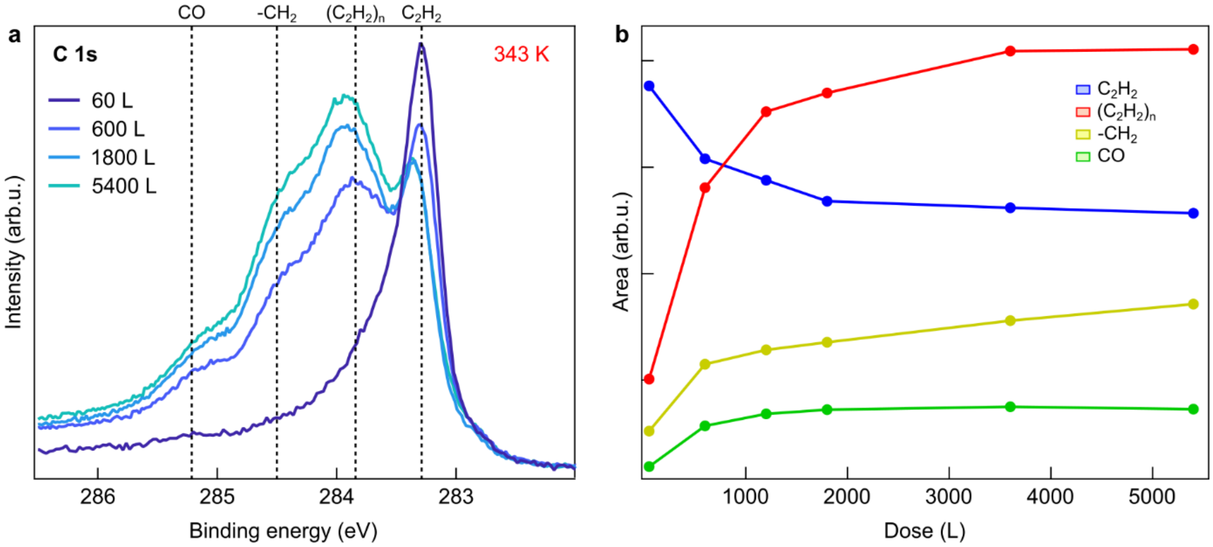

Figure S4. Time evolution of surface carbon species. a) C 1s spectra acquired after different ethylene doses (p = 5×10^{-7} mbar) on Ni(111) at 343 K. b) Areas of the C 1s components plotted as a function of the ethylene dose. After a rapid change occurring upon dosing 1000 L, the surface coverages saturate. No complete conversion of acetylene in polymers has been observed, being likely due to the steric effects which limit the diffusion of the acetylene species.

[1] A. Baraldi, M. Barnaba, B. Brena, D. Cocco, G. Comelli, S. Lizzit, G. Paolucci, R. Rosei, *Journal of Electron Spectroscopy and Related Phenomena* **1995**, *76*, 145–149.

[2] E. Kokkonen, F. Lopes da Silva, M.-H. Mikkelã, N. Johansson, S.-W. Huang, J.-M. Lee, M. Andersson, A. Bartalesi, B. N. Reinecke, K. Handrup, others, *Journal of Synchrotron Radiation* **2021**, *28*, 588–601.

[3] P. J. Flory, *Principles of Polymer Chemistry*, Cornell University Press, **1953**.

[4] L. Grill, S. Hecht, *Nature Chemistry* **2020**, *12*, 115–130.
